# Creation of a free, Internet-accessible database: the Multiple Target Ligand Database

**DOI:** 10.1186/s13321-015-0064-8

**Published:** 2015-04-15

**Authors:** Chao Chen, Yang He, Jianhui Wu, Jinming Zhou

**Affiliations:** North China Institute of Science and Technology, Beijing, China; Institute of Medicinal Biotechnology, Chinese Academy of Medical Science, Beijing, China; Lady Davis Institute for Medical Research, Jewish General Hospital, Montreal, QC Canada

**Keywords:** Polypharmacology, Multiple-target ligands, Database, Drug discovery

## Abstract

**Background:**

Polypharmacology plays an important part in drug discovery, and remains a major challenge in drug development. Identification of the underlying polypharmacology of a drug, as well as development of polypharmacological drugs, have become important issues in the pharmaceutical industry and academia.

**Description:**

Herein, through data mining of the Protein Data Bank (PDB), a free, Internet-accessible database called the Multiple Target Ligand Database (MTLD; www.mtdcadd.com) was constructed. The MTLD contains 1,732 multiple-target ligands (MTLs) which bind to 14,996 binding sites extracted from 12,759 PDB structures. Among MTLs, 222 entries are approved drugs and 1,334 entries are drug-like compounds. The MTLD could be an extremely useful tool in the development of polypharmacological drugs. It also sheds light on the side effects of drugs through anticipation of their multiple functions and similarities in the binding sites of multiple targets. The entire database is free for online searching, browsing, and downloading.

**Conclusion:**

As a crucial expansion of the PDB, increasing numbers of MTLs will be included in the MTLD. Eventually, it will become an efficient platform to obtain useful information on MTLs and their underlying polypharmacology.

## Background

“Polypharmacology” (also termed “drug promiscuity”) refers to the action of a single drug on multiple targets through a single pathway or multiple pathways. This phenomenon has been regarded to be the main cause for the severe adverse effects or toxicities of several drugs approved since the 1990s [1-3]. Based on the exponential growth of molecular data and rapid advances in drug development, evidence suggests that polypharmacology is also important for drug efficacy. For instance, clozapine is the “gold standard” anti-psychotic drug exhibiting beneficial effects *via* complicated interactions with multiple target networks [4]. Several highly efficacious drugs such as salicylate, metformin, or imatinib exhibit enhanced therapeutic efficacy through interactions with multiple targets simultaneously.

In general, it is accepted that the activity towards a single target is not sufficient for a complex disease involving multiple pathogenic factors (e.g., cancer, diabetes mellitus, neurodegenerative syndrome, cardiovascular diseases). Importantly, some of the undesired side effects are due to drugs not hitting their targets, which can confer potential repurposing opportunities for these drugs and provide novel strategies in drug design.

Taken together, polypharmacology plays an important part in drug discovery and remains one of the major challenges in drug development. It opens avenues for rational design of new agents that are more efficient and less toxic than their predecessors [5-10]. Drug discovery using a polypharmacology approach has become a hot topic in the pharmaceutical industry and in academia [5-7,11].

There are hundreds of publicly available databases on drug discovery Protein Data Bank (PDB) [12], DrugBank [13], Kyoto Encyclopedia of Genes and Genomes (KEGG) [14], ZINC [15], Chemical database of European Molecular Biology Laboratory [16], and Therapeutic Target Database [17]. Such databases are key resources that integrate diverse information such as molecular pathways, crystal structures, binding experiments, side effects, and drug targets. Such information is also very useful in prospective drug design using a polypharmacology approach. However, finding information on polypharmacological agents is difficult because of the swathes of information contained in such databases. Thus, development of a novel data-mining method archiving polypharmacological information is needed.

The PDB is a repository of detailed three-dimensional (3D) structural information of proteins and other molecules, including information on the binding between ligands and proteins. It is extremely helpful in the elucidation of ligand promiscuity. Recently, based on the information obtained from the PDB, evidence indicates that similarities in binding sites among multiple proteins and the molecular complexity of a ligand could be reasons for the polypharmacology of drugs [18,19]. As a result, several datasets of multiple-target ligands (MTLs) derived from the PDB have been built by comparing the similarities of binding sites (e.g., Kahraman, Extended Kahraman, Huang) [20,21]. However, the overall entries of MTLs in these datasets are ≤100. Through analyses of ligand promiscuity based on the PDB, an additional two datasets have been generated, containing 164 and 247 entries, respectively [18,19]. However, these datasets do not include all of the potential MTLs in the PDB, and the information in these datasets is not sufficient. Hence, a database containing all of the potential MTLs in the PDB is needed.

Herein, a database termed the Multiple Target Ligand Database (MTLD, www.mtdcadd.com) based on 3D structural data extracted from the PDB has been constructed. The MTLD contains all of the ligands binding to MTLs and sheds light on the side effects through anticipation of their multiple functions as well as the similarities in the binding site of multiple targets. The entire database is free for online searching, browsing, and downloading. Collectively, the MTLD is extremely useful for the development of polypharmacological drugs, and provides various potential candidates for further optimization.

## Construction and content

**Figure 1:**
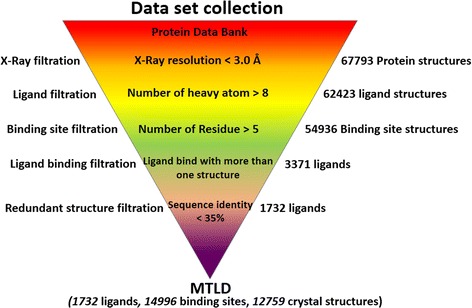
Dataset collection of the MTLD.

Original structural datasets were downloaded from the File Transfer Protocol (FTP) archive (version: December 2012) of the PDB using the script “rsyncPDB.sh”. Datasets were data-mined step-by-step automatically through the command programs written in Perl language (Figure 1).

Atomic structures with a resolution of ≥3.0 Å could be inferred. Hence, X-ray protein structures with a resolution <3.0 Å (67,793 entries) from the PDB were selected for the extraction of ligands and their binding sites. To avoid selection of solvent molecules, ligands containing >8 heavy atoms were extracted from selected PDB files. As a result, 62,423 ligand coordinate files were obtained. Binding sites were defined as all of the protein residues within a radius of 6.0 Å of each atom in binding ligands. Binding sites with >5 residues were outputted, and 54,936 binding sites were extracted, which could bind with 12,138 ligands. Among these ligands, 3,371 ligands were found to bind to more than one PDB structure, and these ligands were chosen for the next filtration. To remove the redundancy of crystal structural entries bound to the same ligand, the sequence identity between protein pairs was restricted to <35%. Eventually, 1,732 MTLs were extracted from the PDB and archived in the MTLD.

Each ligand entry contains five pieces of information. First, the 3D structures of the ligand extracted directly from the known crystal structures are provided. Second, the two-dimensional structure of the ligand that had been converted into “SMILES” format is given. Third, the structures of the binding site that were outputted according to the coordinates of the ligand are detailed. Fourth, the original crystal structures from the PDB to which the ligand binds is given. Last, information on the sequence of the involved proteins downloaded from the PDB or Universal Protein Resource (UniProt; www.uniprot.org) is provided.

Altogether, the MTLD comprises 1,732 MTLs, ≈14.3% of total unduplicated extracted ligands (12,138 entries), which bind with 14,996 binding sites from 12,759 crystal structures. Overall, the MTLD (Table 1) is the most comprehensive, detailed and complete database of MTLs compared with other existing databases on MTLs.

**Table 1:** **Comparison of the MTLD with other datasets of multiple-target ligands**

**Dataset**	**MTL entries**	**PDB structures**	**Accessibility**
Huang	12	143	Yes
Kahraman	9	100	Yes
Extended Kahraman	10	972	Yes
Homogeneous	10	100	Yes
PDB MTL I	164	712	No
PDB MTL II	518	8166	No
***MTLD***	***1732***	***12759***	***Yes***

### Statistical analyses for the MTLD

**Figure 2:**
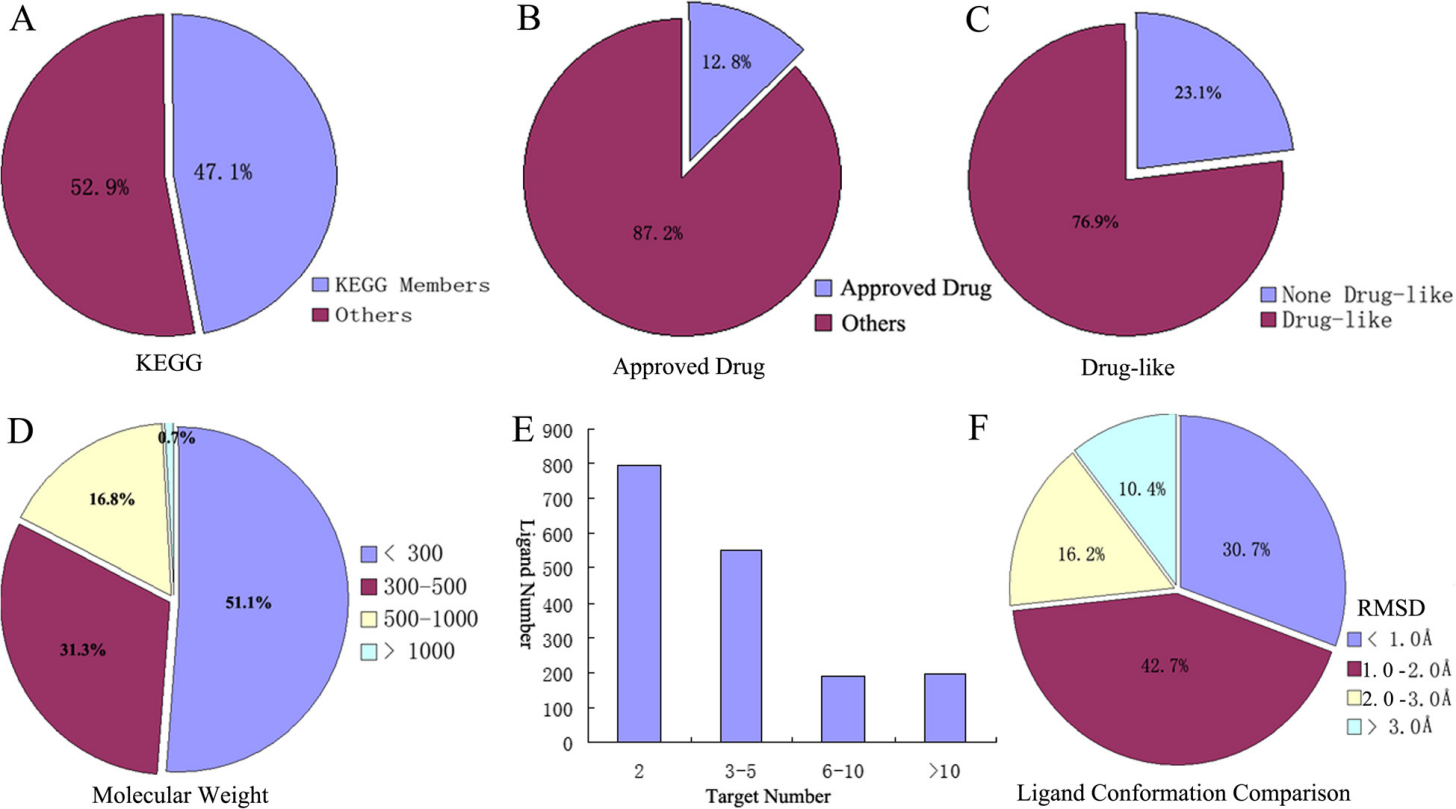
Statistical analyses for entries in the MTLD. **(A)** 815 (47.1%) entries belong to the Kyoto Encyclopedia of Genes and Genomes database; **(B)** 222 (12.8%) entries are approved drugs from the DrugBank database; **(C)** 1334 (76.9%) entries are drug-like compounds according to Lipinski’s rule of five; **(D)** molecular weights of most ligands are ≤500 Da. **(E)** Statistical analyses of the target number of ligands included in the MTLD: 795 ligand entries bind two targets; 551 ligand entries bind 3–5 targets; 189 ligand entries bind 6–10 targets; and 197 ligand entries bind to >10 targets; **(F)** Comparison of the conformation of a ligand bound to different targets. Root-mean-square deviation (RMSD) value was calculated to evaluate the change in conformation: the RMSD value of most ligands was ≤2 Å, indicating a small conformational change.

To better understand constitution of the MTLs in the MTLD, statistical analyses of the MTLD were undertaken (Figure 2). First, the KEGG database (a database of small molecules, biopolymers, and other chemical substances relevant to biological systems) was used to analyse the relationship between MTLD entries and biological processes. In total, 815 MTL entries in the MTLD also belonged to the KEGG database (≈47.1% of overall entries; Figure 2A), which includes various amino acids, saccharides, nucleotides, and lipids. Similarly, in contrast to the known drugs listed in the DrugBank, 222 approved drugs were found in the MTLD (≈12.8% of overall entries; Figure 2B). In particular, by using the module “QuaSAR-Descriptor” included in Molecular Operating Environment (Chemical Computing Group, Montreal, Canada) according to Lipinski's rule of five, 1,334 entries were predicted to be drug-like compounds (≈76.9% of overall entries; Figure 2C). Analyses of the distribution of the molecular weights of MTLs in the MTLD indicated that most of the MTLs had molecular weights <500 Da, and that a very small portion of MTLs had a molecular weight >1000 Da (Figure 2D). Thus, statistical analyses suggested that the MTLD could be highly relevant to biological processes and the action mechanism of drugs.

Among 1,732 MTLs, ≈45.9% ligands (795 entries) were bound to two distinct proteins (Figure 2E), which was lower than the result (65%) reported by Noé Sturm et al. [19]. This result was probably caused by using a different source of datasets when they adopted the sc-PDB (a database derived from the PDB). Notably, 222 ligands were bound to >10 proteins, including approved drugs such as isotretinoin, spermidine, and salicylic acid. The promiscuity of a ligand is related to its conformational flexibility [19]. Hence, analyses of the conformational complexity of the extracted PDB structures for each ligand were conducted through structural alignment using Multiscale Modeling Tools for Structural Biology (Scripps Research Institute, San Diego, CA, USA). The root-mean-square deviation (RMSD) of structure pairs was calculated after the alignment. The maximal RMSD value of the structure pairs was taken to be a criterion of conformational change. Computed RMSD values of most MTLs (1,270 entries, ≈73.3%) were <2.0 Å (Figure 2F), indicating that most of the MTLs could bind to different proteins by adopting a similar conformation. However, further comprehensive analyses are needed to identify the effects of other parameters such as the: molecular size and flexibility of MTLs; number of potential targets; mode of interaction between MTLs and targets.

### Internet interface of the MTLD

The Internet server of the MTLD (mtdcadd.com) is a free, accessible database of MTLs obtained from data mining of the PDB. The Internet server was built using MySQL, Java, Javascript, and HTML languages on a machine with four 2.13-GHz processors. Java and Javascript enable the search function. Java Runtime Environment needs to be installed on the client/customer side. The 3D structures of ligands and proteins were visualized using an open-source Java viewer: Jmol. The structural similarity of ligands was calculated using the FP2 fingerprint through an open-source chemical toolkit: Open Babel.

The MTLD is an easily usable and fully searchable database with many built-in tools. On the MTLD homepage and “About” webpage, a brief introduction of the MTLD is given. The “Download” webpage provides the download option for all data, including approved drugs, KEGG ligands, and some kinase inhibitors. All can be downloaded conveniently. On the “Statistics” webpage, the results of statistical analyses are provided (as mentioned above) and more statistical results will be revealed on this page in the future.

**Figure 3:**
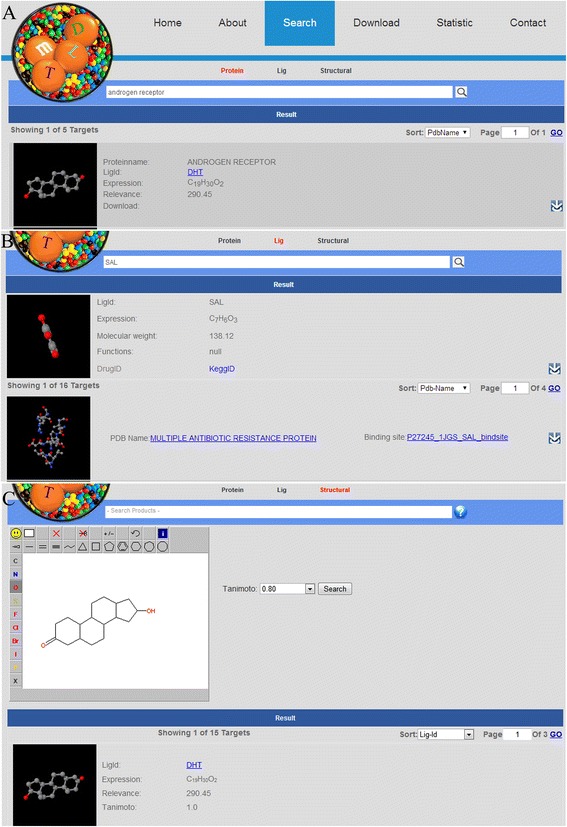
Three examples of searches of MTLs on the MTLD Internet server: **(A)** ligands that bind to the androgen receptor were queried in the “Protein” option; **(B)** ligands with the ligand-ID of “SAL” were queried in the “Lig” option; **(C)** ligands with structural similarity to that of dihydrotestosterone were queried in the “Structural” option.

The link “Search” provides three options. The first is the “Protein” option, which can be searched according to the name, PDB-ID, or UniProt-ID of proteins. For example, a query using the protein name “androgen receptor” was submitted. Five entries were presented in tabular format on the results page showing the: 3D structures of ligands; name, formula, molecular weight of the ligand; ligand-ID of the PDB. One can also proceed to each corresponding webpage of each entry with hyperlinks to other databases such as the PDB, KEGG, DrugBank, and UniProt (Figure 3A). The “Lig” option can be searched by the ligand-ID of the PDB, ligand name, or InChI key. For example, using salicylic acid (ligand-ID: SAL) for searching, 16 non-redundant protein targets binding with it were obtained on the results page (Figure 3B). The “structural” option enables users to draw the queried structures of ligands in the Journal Molecular Editor window. For example, users draw dihydrotestosterone as a query compound with a Tanimoto score cutoff of 0.8 (the Tanimoto score cutoff can be selected from the drop-down menu). Fifteen “hits” were presented in a tabular format on the results page (Figure 3C).

## Discussion

We have demonstrated that the MTLD provides readily accessible information for MTLs (e.g., binding sites extracted from PDB structures, drug-like information, structural similarity of ligands) as well as convenient hyperlinks to databases such as UniProt, DrugBank, KEGG, and the PDB. In addition, redundancy is very common in the PDB. For example, in the PDB, dihydrotestosterone has been found to bind with 37 proteins, which belong to only three targets (Table 2). In such circumstances, the MTLD exhibits the target information of MTLs clearly by filtration of redundant information. However, filtration can result in the loss of some important information, especially for some kinase families, which have very similar amino-acid sequences. In such cases, switching off the “35% Sequence Identity Filtration” option in the search webpage gives the full list of proteins to which a ligand binds.

**Table 2:** **Multiple targets of selected drugs and natural products in the MTLD**

**Ligand (ligand-ID)**	**Target number**	**Targets (PDB-ID)**
Sutent (B49)	4	Mast/stem cell growth factor (3G0E)
		Phosphorylase b kinase gamma (2Y7J)
		Cyclin-dependent kinase 2 (3TI1)
		Tyrosine-protein kinase ITK/TSK (3MIY)
Imatinib (STI)	5	Homo sapiens v-kit Hardy–Zuckerman (1 T46)
		Ribosyldihydronicotinamide dehydrogenase (3FW1)
		Tyrosine-protein kinase SYK (1XBB)
		Mitogen-activated protein kinase 14 (3HEC)
		Bcr-Abl protein
Sorafenib (BAX)	4	B-Raf proto-oncogene serine/threonine-protein (1UWH)
		Vascular endothelial growth factor (4ASD)
		Cyclin-dependent kinase 8 (3RGF)
		Mitogen-activated protein kinase 14 (3GCS)
Dihydrotestosterone (DHT)	3	Sex hormone-binding globulin (1D2S)
		Estradiol 17-beta-dehydrogenase 1 (3KLM)
		Androgen receptor (1I37)
Estradiol (EST)	5	Short chain 3-hydroxyacyl-CoA dehydrogenase (1E6W)
		Sex hormone-binding globulin (1LHU)
		17-beta-hydroxysteroid-dehydrogenase (1FDS)
		Estrogen sulfotransferase (1AQU)
		Estrogen receptor beta (2J7X)
Triiodothyronine (T3)	4	Transthyretin (1SN5)
		Thyroid receptor alpha (THRA) protein (2H79)
		Androgen receptor (2PIV)
		Proliferating cell nuclear antigen (3VKX)
Resveratrol (STL)	7	Sulfotransferase family cytosolic 1B (3CKL)
		Transthyretin (1DVS)
		Myosin-2 heavy chain (3MNQ)
		Leukotriene A-4 hydrolase (3FTS)
		NRH dehydrogenase [quinone] 2 (1SG0)
		Protein (chalcone synthase) (1CGZ)
		Methionine adenosyltransferase 2 subunit (2YDX)
Epigallocatechin gallate (EGCG; KDH)	3	Polymerase subunit PA (4AWM)
		Transthyretin (3NG5)
		Peptidyl-prolyl *cis-trans* isomerase NIMA-inte (3OOB)
Theophylline (TEP)	3	Chitinase
		Apolipoprotein A-I-binding protein
		Pyridoxal kinase
Pioglitazone (P1B)	2	Peroxisome proliferator-activated receptor ga
		Amine oxidase [flavin-containing] b

A crystal structure with a bound ligand does not necessarily mean firm binding. The binding affinity or binding energy of the ligand are important parameters to show if the interaction between the ligand and protein is specific, which can help to judge the “true” target of the ligand. Thus, a link to BindingDB was added on the ligand webpage, and one can find the reported data on binding affinity on the linking page. Moreover, most data on the binding affinity of complexes were not available. Hence, evaluation of the binding free energy using the X-Score method according to the complex coordinate [22] was undertaken, and the value of the binding free energy of the complex shown on the webpage. Furthermore, the crystal structures of some target classes (e.g., kinases) can be obtained much more readily than those of other target classes (e.g., G protein-coupled receptors). In such cases, a bias may be added into the MTLD because the target will not be shown in the database if a crystal structure for that target has not been solved. Further enhancement is needed to try to include such targets into the MTLD.

## Utiliy

Most of the entries in the MTLD are based on drugs. Hence, the MTLD should be useful for developing polypharmacological agents, and may provide potential candidates for further optimization. For example, estrogen receptor-alpha is a drug target for treating breast cancer, and 17-hydroxysteroid dehydrogenase (17HSD1) is a putative target for endocrine therapy of hormone-dependent breast cancer [23]. By searching the MTLD, a ligand, estrogen (ligand-ID: EST) binding to 17HSD1 (PDB-ID: 1FDS) and estrogen receptor (PDB-ID: 3Q95) was found. Thus, polypharmacological drugs that act on both targets could be designed based on the structure of estrogen. Conversely, software called Binding Site Match Maker was developed, which could align binding sites according to their similar physiochemical properties and generate common sites. Binding Site Match Maker could be another option for the design of polypharmacological drugs.

The MTLD can shed light on the multiple mechanisms of action of drugs or natural products. For example, imatinib is an efficacious drug for the treatment of chronic myeloid leukaemia (CML). Imatinib prevents Bcr-Abl protein from exerting its actions in the oncogenic pathway in CML [24]. By searching the MTLD, apart from Bcr-Abl, imatinib was found to bind to mitogen-activated protein kinase 14, ribosyldihydronicotinamide dehydrogenase, tyrosine-protein kinase Syk, and c-Kit kinase. The natural product resveratrol (which is present in red wine) exhibits considerable chemical diversity and biological activities [25]. In the MTLD, resveratrol was found to bind with seven targets. More examples of drugs and natural products are listed in Table 2.

Information obtained from the MTLD can be used to address the mechanism of action of the adverse side effects of drugs. For example, the methylxanthine derivative theophylline (used to treat the symptoms of reversible airflow obstruction) can cause headaches, agitation, and other adverse neuronal side effects. Upon searching the MTLD, pyridoxal kinase was found to be one of the targets of theophylline, which could be a possible underlying mechanism of the neurotoxic effects of theophylline [26]. Likewise, pioglitazone (used for treating type-2 diabetes mellitus) exhibits neuronal side effects, including central nervous system (CNS) depression. Upon searching the MTLD, it was found that pioglitazone can bind to monoamine oxidase B, which may be responsible for CNS depression [27].

Similarities in the binding site have important roles in polypharmacology [18]. Several methodologies have been developed to evaluate similarities in binding sites, such as SiteComp [28] and MultiBind [29]. These methodologies provide useful tools to predict the binding-site similarity of proteins but because of their incompleteness, need to be improved [30]. In such circumstances, the MTLD could provide various binding sites as the training sets or testing sets for the development of novel methodologies for comparisons of binding sites.

## Conclusion

Here we describe development of a comprehensive, Internet-accessible database called the MTLD based on datasets extracted from the PDB. To date, the MTLD comprises 1,732 MTLs that bind to 14,996 binding sites extracted from 12,759 PDB structures. In the MTLD, the 222 entries are approved drugs and 1,334 entries are drug-like compounds. Thus, the MTLD could be extremely helpful for developing polypharmacological drugs and could provide potential candidates for further optimization. Moreover, the MTLD may shed light on the: side effects of drugs; multiple functions of small biological molecules; similarities in binding site of target proteins. As a crucial expansion of the PDB, increasing numbers of MTLs will be included in the MTLD, which will become an efficient platform to obtain useful information on MTLs.

## Availability and requirements

MTLD is freely accessible at http://www.mtdcadd.com. The data in MTLD is free for search, download, and further analysis.

## Abbreviations

PDB: Protein Data Bank
MTLs: Multiple Target Ligands
MTLD: Multiple Target Ligand Database
KEGG: Kyoto Encyclopedia of Genes and Genomes
TTD: Therapeutic target database
DS: Discover studio
RMSD: Root mean square deviation
MMTSB: Multiscale modeling tools for structural biology
ERα: Estrogen receptor α
17-HSD1: 17-hydroxysteroid dehydrogenase type 1
